# Peripheral nerve blocks for primary and secondary headache disorders: review of current evidence and a practical approach

**DOI:** 10.22514/jofph.2026.002

**Published:** 2026-01-12

**Authors:** Sophie McGough, Linford Fernandes, Luis Idrovo

**Affiliations:** ^1^Department of Neurology, Leeds Teaching Hospitals NHS Trust, LS1 3EX Leeds, UK

**Keywords:** Peripheral nerve blocks, Chronic migraine, Corticosteroids, Greater occipital nerve, Cluster headache, Secondary headache disorders

## Abstract

Headache is prevalent, disabling, and a frequent neurological referral in the healthcare system. Clinic-based procedures have evolved in recent years to play an important role in headache medicine, with growing evidence on the safety, tolerability and efficacy of peripheral nerve blocks (PNBs). Despite novel headache therapies, PNBs are still widely used in headache services to treat primary and secondary headache disorders, including cluster headache and other trigemino-autonomic cephalalgias, migraine, occipital neuralgia, and other less frequent headache disorders. We aim to provide an update of the current evidence and a practical approach for delivering the most common PNBs used in clinical practice. We aim to describe PNBs indications, contraindications, injection locations and techniques, drug constituents, and potential pitfalls.

## 1. Introduction

Headache is common and one of the most disabling and undertreated neurological conditions [1, 2]. Peripheral nerve blocks (PNBs) are delivered in acute medical units and outpatient clinics to treat primary and secondary headache disorders. The greater occipital nerve (GON) is commonly targeted, but headache practitioners may target other cervical and distal trigeminal nerve branches [3]. PNBs can reduce the nociceptive input into the trigeminocervical complex modulating central descending inhibitory pain pathways, which can abort acute headache attacks or shorten duration of bouts. PNBs containing local anaesthetics (LA) modulate pain via blocking C- and Aδ nerve fibres. Corticosteroids can be added to LA, although evidence for their efficacy, other than in cluster headache (CH), is scarce [4].

There have been multiple meta-analyses looking at the use of GON blocks in migraine, both for acute and preventive management [5]. A review of the research over the last 5 years has shown increasing evidence that PNBs can be used for a wide variety of headache disorders. The GON block is the most frequently targeted and researched, with good evidence for its use as a transitional preventive therapy in cluster headache and migraine and as a diagnostic and therapeutic tool in occipital neuralgia [6, 7, 8, 9, 10]. In this review, we highlight the evidence for PNBs in primary and secondary headache disorders commonly treated in headache services. Guidance is also provided on the technical and practical aspects of delivering PNBs. The most relevant studies of PNBs in primary and secondary headache disorders, described in this manuscript are summarised in **Supplementary Table 1**.

## 2. Methods

### 2.1 Search strategy

This scoping review outlines the evidence base for PNBs in primary and secondary headache disorders. The authors searched Offshore Vessel Inspection Database (OVID) Medline and PubMed for original research articles published until February 2025. Search terms used were “Greater Occipital Nerve Blocks”, combined with each of the following search terms, “Migraine”, “Cluster headache”, “Primary headache disorders” and “Secondary Headache disorders”. Manuscript titles and abstracts were screened by LF to include manuscripts which were randomised controlled trials (RCTs). For some of the primary and secondary headache disorders with limited or no RCT manuscripts, the largest cohort studies were included. Manuscripts were included if they did not use ultrasound guided nerve blocks, and focused on distal greater occipital nerve blocks. The authors excluded manuscripts which researched sub-occipital or cervical nerve blocks. This distinction was made because sub-occipital nerve blocks, cervical nerve blocks, and ultrasound guided nerve blocks are delivered primarily by pain specialists in the UK and neurologists deliver distal greater occipital nerve blocks based on anatomical landmarks. The studies referenced in this review are outlined in **Supplementary Table 1**.

### 2.2 PNBs in migraine

Randomised controlled trials (RCTs) have shown the efficacy of GON blocks in episodic and chronic migraine. RCTs using GON block injections are problematic to design due to possible inadequate blinding of patients with a procedure where the use of local anaesthetic is part of the treatment protocol [10]. Despite these limitations, an evidence base has been established over the last few years.

In a RCT of episodic migraine patients, a single GON block with triamcinolone and lidocaine reduced the frequency of migraine attacks in the four weeks following the injection, but had no difference to placebo on the duration and severity of the migraine headache [11]. Bilateral GON blocks given weekly for three weeks, were shown to reduce the frequency of episodic migraine at two months [12]. As an acute treatment for migraine, bupivacaine GON blocks provided freedom from pain in up to 31% of patients compared with placebo and was as effective as intravenous metoclopramide and Non-steroidal anti-inflammatory drugs (NSAIDs) [13, 14]. However, a RCT of GON block with bupivacaine compared to intravenous metoclopramide for acute headache treatment in the emergency department was shown to be equivalent [15]. PNBs targeting the supraorbital nerve (SON) and GON have also shown to be superior to the use of SON blocks alone in acute migraine management [16]. Interestingly, in patients with acute migraine aura, bupivacaine GON blocks completely resolved the aura in 50% of patients with no recurrence of aura in the following week, and in chronic migraine, the same GON blocks showed significant improvement in pain scores at four weeks [17, 18].

In chronic migraine, GON blocks with bupivacaine were efficacious in short-term preventative treatment, with the addition of triamcinolone to the GON block not showing any additional significant benefit to the use of bupivacaine alone [19, 20, 21]. When given weekly for four weeks, bupivacaine GON blocks significantly reduced headache frequency and pain at up to three months of follow-up [22]. In another RCT, the use of triamcinolone and lidocaine GON blocks resulted in reduction of pain severity and frequency as well as use of analgesics up to two months after the intervention [23]. GON blocks with methylprednisolone and lidocaine/bupivacaine when combined with lesser occipital nerve (LON), supratrochlear nerve (STN), SON, and auriculotemporal nerve (ATN) blocks helped reduce headache attack frequency in up to two thirds of chronic migraine patients in an open label and a prospective non-randomised cohort in the UK [3, 8]. In chronic migraine patients who respond to GON blocks, the addition of prophylactic medication has not shown any further improvement beyond that provided by the GON block alone [24]. However, in a RCT of patients receiving topiramate monotherapy, monthly GON blocks were more effective in reducing monthly migraine days than topiramate, providing evidence for the use of these blocks in patients refractory to medication treatment [25].

In chronic migraine patients with medication overuse headache, GON blocks provided better outcomes compared with standard detoxification regimens which involve medication withdrawal, and perhaps it could be a more efficient transitional strategy to help patients withdraw acute medications including triptans and NSAIDs [26, 27]. A RCT of GON blocks with triamcinolone given to patients with medication overuse headaches in addition to medication withdrawal showed that detoxification with GON blocks resulted in better outcomes [28]. In patients with migraine, either as an acute, transitional, or preventive treatment, the evidence of adding corticosteroids to the injection preparation to improve patient’s outcome is scarce [29, 30, 31]. However, considering the methodological difficulties in RCTs, Dilli *et al*. [21] showed that both the active and placebo arm were equally effective and well tolerated. Moreover, a few open label studies, including our own experience, suggest that adding corticosteroids is safe, well tolerated, and theoretically could prolong the anaesthetic effect as seen in other PNB studies [32, 33].

### 2.3 PNBs in cluster headache

The use of GON blocks in episodic and chronic cluster headache as a preventive treatment is well established in headache clinics [33, 34, 35, 36]. More recently, the 2023 European Academy of Neurology Guidelines on the treatment of cluster headache recommended the use of GON block with the use of steroids in treatment of cluster headaches [37]. A RCT of episodic cluster headache found that methylprednisolone and lidocaine GON blocks significantly reduced weekly attack frequency at four weeks post injection compared with placebo [38]. RCTs of episodic and chronic cluster headaches have additionally demonstrated that addition of GON blocks to oral verapamil resulted in a reduction in the number of daily attacks in the week following the injection and up to four weeks in some patients [39, 40].

Observational cohort studies provide evidence that a single GON block can reduce attack frequency in the week following the injection, which allows GON blocks a role in the acute treatment of a cluster of attacks in these patients whilst oral medication is being optimised [33, 34, 41].

### 2.4 PNBs in other trigemino autonomic cephalalgias (TACs)

Although the evidence is limited, headache practitioners commonly use GON blocks with and without the addition of trigeminal nerve blocks (multiple cranial nerve approach) as a transitional preventive or co-adjuvant treatment of other TACs, such as hemicrania continua (HC), Paroxysmal hemicrania (PH) and SUNCT-SUNA (Short-lasting Unilateral Neuralgiform attacks with Conjunctival injection and Tearing, and Short-lasting Unilateral Neuralgiform attacks with Cranial Autonomic symptoms) [8, 42, 43, 44]. Lambru *et al*. [43] reported that in his cohort of 78 patients with SUNCT-SUNA, 66 received a unilateral GON blocks, whereas 12 patients received bilateral injections in view of their unilateral side-alternating attacks. Headache improvement after the injection was reported by 37% (n = 29/78) of patients lasting for a mean of 38.4 ± 34.7 days. Some headache practitioners advocate the use of ipsilateral greater and lesser occipital nerve (LON) blocks as first-line treatment in patients with TACs if medications are not well tolerated, *i.e.*, lamotrigine in SUNCT-SUNA and if indomethacin is not tolerated or contraindicated in patients with suspected Indomethacin responsive headaches—hemicrania continua and paroxysmal hemicrania [8, 43, 44].

### 2.5 PNBs for secondary headache disorders

There is robust evidence to support the use of GON blocks in cervicogenic headache with and without associated occipital neuralgia [9, 45]. In a RCT of patients with cervicogenic headaches, occipital nerve blockade significantly relieved cervicogenic headache and associated symptoms at two weeks following the injection [46]. Another RCT of patients with occipital neuralgia and cervicogenic headaches showed significant reduction in headache days and analgesia use when compared with baseline after receiving ultrasound guided GON blocks at the level of C2 [47]. In a cohort study, GON blocks given via sub compartmental technique showed longer headache relief, whilst another RCT of patient with cervicogenic headache showed improvement with both GON and Cervical level 2/3 nerve blocks [48, 49]. Furthermore, the use of repetitive GON blocks resulted in prolonged pain relief lasting up to six months in patients with cervicogenic headache [9]. There is less evidence for PNBs for secondary headaches but it’s use in post dural puncture headaches has been studied over the last few years. RCTs of post dural puncture headaches have consistently shown that GON blocks significantly improve pain scores and at up to 24 hours post block compared with standard treatment alone [50, 51, 52].

In patients with post dural puncture headaches following caesarean section under spinal anaesthesia, a RCT has shown that the use of dexamethasone and lidocaine delivered to suboccipital muscles significantly improved pain scores at 24 hours compared with placebo [53]. A further cohort study in patients after spinal anaesthesia support these findings with improvement in pain scores at 24 hours after a GON block [54].

There are also case reports and smaller case series suggesting a role for GON blocks in the management of other headache disorders, including spontaneous intracranial hypotension, subarachnoid haemorrhage, and post-traumatic headaches [55, 56, 57, 58]. However, whilst these studies and reports demonstrate improvement in pain scores and clinical outcomes in these patients, further studies with more patients and RCTs are needed for these headache disorders. Unlike migraine and cluster headache, these secondary headaches disorders are relatively less prevalent, making it more difficult to study systematically.

### 2.6 Safety profile of PNBs

PNBs are generally safe and well-tolerated. The most common side effects include pain at injection site, dizziness, and nausea [42]. A systematic review of GON blocks found the only irreversible adverse effects to be avascular necrosis of the hip, although causality in this case was not established, and persistent alopecia very rarely [59]. Further meta-analysis comparing local anaesthetic only blocks to placebo found no significant difference in adverse events [60].

Local anaesthetic myotoxicity and systemic toxicity are rare complications of the use of local anaesthetics and can be mitigated through the rationing of the total anaesthetic dose used at a single timepoint and minimising injecting into local blood vessels [61, 62]. Injector training and anatomical localisation are important factors in minimising these risks. Local anesthetic systemic toxicity (LAST) is a very rare but potentially life-threatening complication of local anesthetic administration, most notably after the use of Bupivacaine compared with Lidocaine and affecting the central nervous and cardiovascular systems [63]. Neurological symptoms usually include perioral paresthesias, a metallic taste, tinnitus, and agitation, followed by seizures, and ultimately neurologic depression with respiratory arrest and eventually coma if unsupported. The incidence of LAST is variable and recent reviews of case reports and registries have estimated its incidence as low as 0.27 per 1000 and as high as 1.8 per 1000 peripheral nerve blocks [64].

A RCT which compared occipital nerve block to acetaminophen and caffeine for headache in pregnancy found no difference in birth weight or mode of delivery, and also found that the nerve block led to a lower pain rating within one hour [65]. Lidocaine-only nerve blocks are considered safe in pregnancy [42].

There are minimal absolute contraindications to performing a cranial nerve block; however they do include allergic reaction to local anaesthetic, and open skull defect. If there is an anaesthetic allergy then the patient can receive a corticosteroids-only block, however this would be limited to only GON blocks. Localised bleeding from the injection site can occur, so care needs to be taken if patients are on anti-platelet or anti-thrombotic medication, and the local site should be pressed for a few minutes following injection [66, 67].

When using a nerve block with steroids, there should also be an awareness of the steroid side effects. Local effects of corticosteroid injection can include alopecia, atrophy, and hypopigmentation [68, 69]. The more systemic effects also need to be considered, and they can include hyperglycemia in patients with diabetes, candidiasis, and Cushing’s disease [69]. It is possible that methylprednisolone has an enhanced safety profile over other corticosteroids as no irreversible adverse effects were reported in its use in a systematic review [59].

### 2.7 Peripheral nerve blocks: technical aspects

Peripheral nerve blocks (PNBs) are increasingly utilized as a therapeutic tool for various headache disorders, including cluster headache (CH), migraine, and other primary and secondary headache disorders [42]. Recommendations and practitioner’s surveys from both the American Headache Society, the Spanish Headache Study Group, and British Association for the Study of headache (BASH) have shaped clinical guidelines for the indications, contraindications, and technical aspects for the administration of PNBs based on an evidence base approach [5, 6, 7, 70]. The constituents of these blocks are contingent upon factors such as availability, clinician preference, and institutional protocols. Local anesthetics (LAs) exert their analgesic effects by inhibiting the conduction of nociceptive signals through nerve fibres, primarily through the reversible blockade of sodium channels targeting C- and Aδ fibres, which are integral to the transmission of pain [4]. While corticosteroids are occasionally added to LAs in this context, the evidence supporting their efficacy—beyond their use in cluster headache—remains limited [71]. Interestingly, despite their widespread use, there is currently no international consensus regarding a standardized technical approach for the delivery of PNBs for headache disorders [72, 73, 74, 75, 76]. The nerve block constituents we use in our centre are outlined in Table 1 (Ref. [42]).

**Table 1.** Nerve block constituents. Adapted with permission from Fernandes *et al*. [42] Practical Neurology, 2021.

Nerve	Constituents per injection (volume injected, mL)	Constituents (anaesthetic only-volume ratio)
Greater Occipital	Methylprednisolone 40 mg/mL* (2 mL) Lidocaine 2%** (1 mL) Bupivacaine 0.5%*** (1 mL) Total injection volume = 4 mL	Methylprednisolone can be omitted and volume made up with lidocaine and/or bupivacaine If a combination of the both lidocaine and bupivacaine is used, the recommended volume ratio (lidocaine/bupivacaine) is 1:1–1:3
Lesser Occipital	Lidocaine 2% (1 mL) Bupivacaine 0.5% (1 mL) Total injection volume = 2 mL	
Auriculotemporal		
Supraorbital	Lidocaine 2% (0.5 mL) Bupivacaine 0.5% (0.5 mL) Total injection volume = 1 mL	
Supratrochlear		

*Methylprednisolone acetate maximum dose 160 mg per sitting, **Lidocaine maximum dose: 4.5 mg/kg, not to exceed 300 mg per dose (without vasoconstrictor), ***Bupivacaine maximum dose: 2.5 mg/kg, not to exceed 175 mg per dose (without vasoconstrictor).

A comprehensive understanding of the anatomy and reference points of the occipital and superficial trigeminal nerve branches is key for the effective and safe performance of PNBs. Knowledge of these anatomical structures is essential not only for maximizing the therapeutic efficacy, but also for mitigating potential complications, such as nerve injury, haemorrhage, or inadvertent arterial injection of the anesthetic agent. Headache patients frequently report their pain localized to the forehead, retro-orbital region, temples, occipital area, and upper cervical regions [42]. The forehead and upper periocular areas are predominantly innervated by peripheral branches of the ophthalmic division (V1) of the trigeminal nerve, including the supraorbital and supratrochlear nerves. The auriculotemporal nerve, a branch of the mandibular division (V3), innervates the temples, while the occipital and upper cervical regions receive innervation from the C2/C3 posterior cervical branches, including the greater, lesser, and third occipital nerves.

Once a patient has been deemed suitable for a PNB, it could be beneficial to provide a graphic representation of the relevant peripheral cranial nerve to be targeted, thereby facilitating patient understanding. We have previously produced and published illustrated videos of PNB administration as an aid for healthcare practitioners [42]. The informed consent process should comprehensively explain the risks associated with the procedure, including potential complications, such as infection, bleeding at the injection site, and procedural discomfort. PNBs are contraindicated at sites with prior surgical interventions, such as previous burr holes or craniotomy, due to the risk of inadvertent anesthetic infiltration into the central nervous system. Additionally, blocks should generally be avoided in patients with implanted devices, such as nerve stimulators or shunts, though in rare circumstances, they may be considered with appropriate expertise and informed consent regarding the potential risks.

Following consent, patients should be positioned either supine on an examination table or seated, depending on the specific nerve to be blocked and patients’ preferences. Pre-procedural recommendations often include ensuring the patient is adequately hydrated and has eaten to reduce the risk of vasovagal episodes. For patients with prior presyncope or syncopal episodes during administration of injectable treatments, we suggest the patient to assume a lateral decubitus position for GON blocks to avoid sudden drop of blood pressure and dizzy spells. Clinicians must rigorously confirm patient identity and the targeted injection site prior to initiating the procedure, ensuring compliance with local safety protocols.

Patients will typically experience localized numbness in the dermatome of the injected nerve within minutes of the procedure, which serves as an indicator of successful nerve infiltration; however there is no correlation between having an immediate anaesthetic effect and short- or long-term treatment efficacy.

It is important to note that corticosteroids are commonly incorporated into GON blocks, though some centres may use them for LON blocks as well. However, their use in trigeminal nerve blocks is generally discouraged due to the risk of unwanted cosmetic side effects, and lipoatrophy. Additionally, the systemic effects of corticosteroids, including iatrogenic Cushing’s syndrome, have been documented in both the literature and anecdotal reports.

#### 2.7.1 Greater occipital nerve (GON) blocks

The GON, arising from the medial branch of the dorsal primary ramus of the second cervical nerve, innervates the posterior scalp extending to the vertex. Anatomically, it is located approximately one-third of the distance between the occipital protuberance (inion) and the mastoid process, typically 2 cm lateral and 1.5–2.0 cm inferior to the inion. Careful attention must be paid to the occipital artery, which typically runs lateral to the GON. The injection technique involves positioning the patient with the head slightly flexed, with the clinician standing behind. A 25-gauge needle is inserted perpendicularly to the skin until firm resistance is encountered, indicating the needle has reached the periosteum. Aspiration is performed to rule out arterial injection, followed by a fan-like distribution of the anesthetic solution.

#### 2.7.2 Lesser occipital nerve (LON) blocks

The LON arises from the ventral rami of the second and third cervical nerves and innervates the lateral posterior scalp. It is located approximately two-thirds of the distance between the inion and mastoid process. The injection procedure mirrors that of the GON, with the patient in a seated position and the clinician standing behind. After locating the LON, the needle is inserted perpendicularly, and the anesthetic solution is injected following aspiration to confirm no arterial flashback.

#### 2.7.3 Supratrochlear and supraorbital nerve blocks

Both the supratrochlear and supraorbital nerves, branches of the ophthalmic division (V1) of the trigeminal nerve, innervate the forehead and anterior scalp. These nerves are superficially located near the supraorbital ridge. The supratrochlear nerve can be blocked by inserting the needle lateral to the procerus muscle, just above the eyebrow, while the supraorbital nerve is blocked just above the supraorbital notch. A 30-gauge needle is used for both, with aspirations to confirm correct needle placement prior to injection.

#### 2.7.4 Auriculotemporal nerve blocks

The auriculotemporal nerve, arising from the mandibular division (V3) of the trigeminal nerve, innervates the temporal region and the temporomandibular joint. Its superficial branches are located anterior to the tragus, making this area the target for injection. The injection technique involves inserting a 30-gauge needle into the subcutaneous tissue, ensuring proper aspiration before injecting the anesthetic solution.

#### 2.7.5 Infraorbital nerve blocks

The infraorbital nerve is a terminal branch of the maxillary nerve (V2). It emerges from the infraorbital foramen and provides sensory innervation to the skin of the lower eyelid, cheek, wing of the nose, and upper lip. This nerve is blocked at its exit point from the infraorbital foramen. This foramen may be identified 6 mm below the infraorbital rim and 25 mm from the midline. With the patient supine, the doctor remains on the same side as the nerve targeted for the injection. We recommend using a 2 to 5 mL syringe and a 30-gauge needle. The needle is angled medially 45 degrees and slightly upward next, 0.5 to 1 mL of anaesthetic solution is injected. Injecting excessive volumes of solution or angling the needle too sharply may result in orbital diffusion of the anaesthetic, a situation best avoided.

## 3. Limitations

The remit of this review was on the use of GON blocks for headache when administered using anatomical landmarks. There is evidence base on the use of ultrasound guidance for the delivery of proximal GON blocks and sub-occipital nerve blocks for headaches. We did not review the studies investigating GON blocks administered through ultrasound guidance. Future studies should compare the use of ultrasound guided proximal GON block delivery with GON blocks delivered distally on headache outcomes.

## 4. Conclusions

Peripheral nerve blocks are effective in the acute, transitional, and preventative management of headache disorders. It is still difficult to identify those who will respond best, but these procedures allow an interventional approach for those with troublesome, acute, and refractory primary and secondary headaches. Neurologists can administer these blocks as a day case procedure, in clinic or the emergency department, where quick pain relief can provide a satisfactory outcome and avoid hospital admissions. Despite its widespread use, an international expert consensus is still lacking and may well guide headache practitioners in selecting the correct candidate patient, the standardized interventional technique, the type, dosage, and volume of the pharmacological agents to be used, defining the optimum intervals for repeat procedures, and to determine standardized treatment and patient outcomes.

## Supplementary Material

Supplementary material associated with this article can be found, in the online version, at https://files.jofph.com/ files/article/2010553951928107008/attachment/ Supplementary%20material.docx.


