# Supplementary material for: Peripheral nerve blocks for primary and secondary headache disorders: review of current evidence and a practical approach
Source: J Oral Facial Pain Headache. 2026 Jan 12;40(1):25–31. doi: 10.22514/jofph.2026.002 (PMC12853162; doi:10.22514/jofph.2026.002)
Supplement: Supplementary file 1 [file Supplementary-material.docx]

Supplementary material

Supplementary Table 1. Outline of PNBs studies in primary and secondary headache disorders.

| Author | | Type of study | Study population | Type of headache | Study outcomes |
| --- | --- | --- | --- | --- | --- |
| Migraine | | | | | |
|  | Dilli *et al.* [21] (2015) | RCT | 63 patients (30 placebo *vs.* 33 treatment) | Episodic or chronic migraine | Results: GON block with steroid and LA did not show any reduction in frequency of headache days, or acute medication use when compared to placebo. Both groups had 30% of patients with at least a 50% reduction in the frequency of headache days. |
|  | Ashkenazi *et al.* [19] (2008) | RCT | 37 patients (18 LA alone *vs.* 19 LA + steroid) | Chronic migraine | Results: Both groups had significant and rapid relief of headache and neck pain severity.  No significant difference in mean headache severity, mean neck pain severity or duration of being headache free in either group. |
|  | Cuadrado *et al.* [20] (2017) | RCT | 36 patients (18 treatment *vs.* 18 normal saline) | Chronic migraine | Results: Anaesthetic block superior to placebo in reducing the number of days per week with both moderate-or-severe headache, or headache of any intensity. |
|  | Gul *et al.* [22] (2017) | RCT | 44 patients (22 LA *vs.* 22 saline) | Chronic migraine | Results: Repeated block with bupivacaine showed significant decrease in frequency of headache and Visual Analogue Score (VAS) scores at first, second, third month of follow up. Placebo had decrease in headache days and VAS score only in the first month. |
|  | Karadas *et al.* [26] (2017) | RCT | 105 patients (35 control, 35 single GONB, 35 3-stage GONB) | Triptan overuse headache | Results: Both single GONB and repeated GONB reduced migraine attack frequency, attack duration, and decreased the severity of pain. Repeated GONB was superior to single GON and medication withdrawal alone in reducing headache frequency at 4 months. |
|  | Kashipazha *et al.* [23] (2014) | RCT | 48 patients (24 intervention *vs.* 24 control) | Chronic migraine | Results: Both groups led to significant reduction in pain severity, pain frequency, and analgesic use. No significant differences found between the two groups. |
|  | Palamar *et al.* [18] (2015) | RCT | 23 patients (11 intervention *vs.* 12 placebo) | Migraine without aura | Results: At 1 week both groups had decrease in headache intensity (VAS score). At 4 weeks significant improvement observed only in the bupivacaine group. |
|  | Friedman *et al.* [15] (2020) | RCT | 99 patients (51 GONB *vs.* 48 metoclopramide) | Acute migraine | Results: IV metoclopramide led to a larger mean pain improvement, greater sustained headache relief and reduced need for rescue medication when compared to GONB. |
|  | Hokenek *et al.* [16] (2021) | RCT | 128 patients (30 group 1, 28 group 2, 43 group 3, 27 group 4) | Acute migraine | Results: GON, SON, and combined block group had significant improvement in VAS scores 120 minute after treatment compared to placebo group. Change in SON group for VAS score was less of that GON and combined group. No significant difference found between GON and combined group. |
|  | Chowdhury *et al.* [25] (2022) | RCT | 121 patients (41 group A, 39 group B, 37 group C) | Chronic migraine | Results: Combination treatment of topiramate with monthly injections of greater occipital nerve block (with LA alone or steroid added) were more effective in reducing monthly migraine days in chronic migraine than topiramate monotherapy. Effect sustained until 3 months. Both the GONB treatments led to reduced headache impact and disability compared to topiramate monotherapy. |
|  | Malekian *et al.* [11] (2022) | RCT | 55 patients (10 group 1, 16 group 2, 13 group 3, 16 group 4) | Episodic migraine | Results: All four groups had significant improvement in mean severity and duration of headaches, with no injection being superior to the placebo. Groups receiving a block with local anaesthetic had significantly less headaches compared to saline or steroid alone. |
|  | Chowdhury *et al.* [73] (2023) | RCT | 44 patients (22 intervention *vs.* 22 placebo) | Chronic migraine | Results used: Lidocaine blocks were superior to placebo in decreasing the average number of headache and migraine days. |
|  | Gursoy *et al.* [74] (2024) | RCT | 66 patients (34 US-guided *vs.* 32 landmark based) | Chronic migraine | Results: US-guided GON block technique resulted in lower VAS scores, shorter durations of pain, lower frequencies of attack, and lower use of analgesics compared to the landmark-based technique. |
|  | Vanderpol *et al.* [75] (2024) | RCT | 60 patients (27 supine, 33 sitting) | Chronic migraine | Results: HIT-6 score significantly improved following the block at day 30 and day 90 in both groups. No significant difference in HIT-6 scores with patient positioning. |
|  | Ozer *et al.* [12] (2019) | RCT | 71 patients | Episodic and chronic migraine | Results: GON and SON blockade with lidocaine led to a significant decrease in headache days and lower VAS score after 2 months compared to placebo. |
|  | Korucu *et al.* [14] (2018) | RCT | 60 patients (20 in each group) | Acute migraine attack | Results: GON blockade was as effective as an IV dexketoprofen + metoclopramide treatment and superior to a placebo in reducing pain severity score 45 minutes following treatment. |
|  | Friedman *et al.* [13] (2018) | RCT | 28 patients (15 sham injection, 13 GONB) | Acute migraine | Results: Greater rates of freedom from headache achieved by GONB at 30 minutes compared to sham. GONB also more likely to achieve sustained headache relief. |
|  | Cuadrado *et al.* [17] (2017) | Cohort study | 18 patients, 22 auras treated | Prolonged migraine aura (>2 hours) | Results: 50% showed complete response with no aura recurrence in week following. 27% reported >50% improvement. |
|  | İnan *et al.* [24] (2016) | Retrospective study | 78 patients (25 medication + GONB *vs.* 53 GONB only) | Chronic migraine | Results: Both groups had significant decrease in headache attack frequency, headache duration, and headache severity. No statistically significant difference between the two groups. |
|  | Tepe *et al.* [27] (2021) | Retrospective study | 82 patients (41 bilateral GONB *vs.* 41 GON + SON block) | Chronic medication overuse headache | Results: Both groups led to significant reduction in headache parameters. However, the numerical rating score (NRS) score, analgesic intake, number of painful days, and pain duration significantly improved in the GON block added SON block group compared to GONB alone. |
|  | Arab *et al.* [28] (2022) | RCT | 54 patients (27 each group) | Medication overuse headache | Results: Both programs were effective in completing detoxification. Detoxification with GONB resulted in better outcomes, with improved frequency, duration, and severity of headache. |
|  | Bushman *et al.* [65] (2023) | RCT | 62 patients (31 intervention *vs.* 31 standard care) | Pregnant patients with headache | Results: Headache improvement to visual rating scale score of 3 or lower, was not significantly different between groups. Nerve block resulted in lower visual rating scale scores at 1 hour compared to standard care. |
| Cluster Headache | | | | | |
|  | Chowdhury *et al.* [38] (2024) | RCT | 39 patients (19 active, 20 placebo) | Episodic cluster headache | Results: GONB with methylprednisolone and lignocaine significantly reduced the weekly attack frequency from baseline to Week 1 through Week 4 compared to a placebo. |
|  | Gaul *et al.* [41] (2013) | Prospective study | 101 patients | Chronic and episodic cluster headache | Results used: More than 80% had complete or partial response following the block. There was a significant reduction in attack frequency and attack duration. |
|  | Abu Bakar *et al.* [33] (2013) | Prospective study | 83 patients | Chronic cluster headache | Results: A positive response was observed in 71% of patients, 51% were pain free, whilst 21% had a partial benefit. Benefit lasted a median of 18 days. |
|  | Leroux *et al.* [40] (2011) | RCT | 43 patients (21 cortivazol *vs.* 22 placebo) | Episode and chronic cluster headache | Results: Repeated suboccipital injection with steroid was found to be effective compared to placebo, leading to fewer daily attacks in the first 15 days following treatment. |
|  | Ambrosini *et al.* [39] (2005) | RCT | 23 patients (13 treatment *vs.* 10 placebo) | Episodic and chronic cluster headache | Results: Steroid injection was significantly more effective than control in leading to attack-free periods (85% compared to 0%), with this effect being sustained for 4 weeks for 60% in the steroid group. |
| Other Primary Headache Disorders | | | | | |
|  | Juskys *et al.* [76] (2018) | Prospective study | 44 patients (29 GONB, 15 GONB + LONB) | Occipital neuralgia | Results: 95% of patients showed satisfactory results for at least 6 months. The mean VAS scores improved 24 hours after injection and remained low at 6 months. No difference found with the local anaesthetic used, or the pain group targeted. |
|  | Naja *et al.* [9] (2006) | Prospective study | 47 patients | Cervicogenic headache | Results: 95% achieved a 6-month pain-free period and stopped medication completely, with 87% of patients requiring more than 1 injection to achieve this. |
|  | Naja *et al.* [46] (2006) | RCT | 47 patients (23 placebo, 24 anaesthetic blockade) | Cervicogenic headache | Results: Patients receiving anaesthetic blockade had decreased analgesic consumption compared to placebo over a 2-week period. VAS scores reduced by 50% of basal values in anaesthetic block. Duration of headache, and associated headache symptoms were also significantly reduced in anaesthetic group. |
|  | Kissoon *et al.* [47] (2022) | RCT | 32 patients (16 in each group) | Occipital neuralgia/cervicogenic headache | Results: Both groups had significant decrease in HIT-6.  The ultrasound-guided GONB had significant decrease in numeric rating scale (NRS) scores from baseline compared with the landmark-based group at 30 minutes and 4-weeks. |
|  | Inan *et al.* [48] (2001) | RCT | 28 patients (14 GON, 14 C2/C3) | Cervicogenic headache | Results: In both groups a considerable decrease in the frequency and degree of pain was seen in the first week after the diagnostic block, in the first week after the first therapeutic block, and in the first and second months following the second therapeutic block.  Reduced pain frequency in the first week following the first therapeutic block seen in C2/C3 group compared to GON group. Otherwise, no significant difference between the two groups. |
|  | Lauretti *et al.* [49] (2015) | RCT | 30 patients (10 in each group) | Cervicogenic headache | Results: The sub-compartmental technique resulted in at least 24 weeks of analgesia, compared to 2 weeks for the classical technique. There was no significant difference in outcomes with the different volumes used with the sub-compartmental technique. |
| Secondary Headache Disorders | | | | | |
|  | Elsayed *et al.* [50] (2023) | RCT | 50 patients (25 each group) | PDPH | Results: Both groups had significantly lower sitting and lying Numeric Rating scale (NRS-11) scores up to 48 hours following intervention. Proximal block led to significantly lower sitting NRS-11 compared to distal blocks. Proximal block also led to significantly less analgesic consumption. |
|  | Abdelraouf *et al.* [53] (2019) | RCT | 90 patients (45 in each group) | PDPH | Results: Intervention group led to a significant decrease in VAS score at 24 hours compared to control at all post-injection time points up to 24 hours. Intervention also led to improved nausea symptoms, and less need for result analgesia. |
|  | Akyol *et al.* [54] (2015) | Retrospective study | 21 patients | PDPH | Results: Mean VAS scores were significantly improved 24 hours following intervention, no matter pre-block VAS score. 100% of patients with pre-block VAS score between 4–6 were considered to have recovered from headache (VAS score of 1). However, only 1 (11.1%) of patients with pre-block VAS score between 7–9 recovered. |
|  | Mostafa Mohamed *et al.* [51] (2019) | RCT | 50 patients (25 each group) | PDPH | Results: GONB group had significant improvement in headache intensity up to 12 hours following injection compared to the control group. GONB group also had less need for oral analgesia. |
|  | Naja *et al.* [52] (2009) | RCT | 47 patients (23 control, 24 block) | PDPH | Results: Patients receiving GONB had significantly lower VAS scores compared to control. The block group also consumed significantly less analgesics and were discharged from hospital significantly earlier. Complete pain relief was achieved in 68.4% of block patients after 1 to 2 blocks. |

PDPH, post dural puncture headache; RCT, randomised controlled trial; GON, Greater occipital nerve; GONB, Greater occipital nerve block; LON, Lesser occipital nerve; SON, Supraorbital Nerve; VAS, Visual Analogue Score; LA, Local Anaesthetic; LONB, Lesser occipital nerve block; IV, intravenous; HIT, Headache Impact Test; US, ultrasound.
